# Novel optimization strategy for tannase production through a modified solid-state fermentation system

**DOI:** 10.1186/s13068-018-1093-0

**Published:** 2018-04-02

**Authors:** Changzheng Wu, Feng Zhang, Lijun Li, Zhedong Jiang, Hui Ni, Anfeng Xiao

**Affiliations:** 1 College of Food and Biological Engineering, Jimei University, Xiamen, 361021 China; 2 Fujian Provincial Key Laboratory of Food Microbiology and Enzyme Engineering, Xiamen, 361021 Fujian China; 3 Fujian Provincial Engineering Technology Research Center of Marine Functional Food, Xiamen, 361021 Fujian China; 4 Xiamen Key Laboratory of Marine Functional Food, Xiamen, 361021 China

**Keywords:** Biomass, Solid-state fermentation, Tannase, *Aspergillus tubingensis*

## Abstract

**Background:**

High amounts of insoluble substrates exist in the traditional solid-state fermentation (SSF) system. The presence of these substrates complicates the determination of microbial biomass. Thus, enzyme activity is used as the sole index for the optimization of the traditional SSF system, and the relationship between microbial growth and enzyme synthesis is always ignored. This study was conducted to address this deficiency. All soluble nutrients from tea stalk were extracted using water. The aqueous extract was then mixed with polyurethane sponge to establish a modified SSF system, which was then used to conduct tannase production. With this system, biomass, enzyme activity, and enzyme productivity could be measured rationally and accurately. Thus, the association between biomass and enzyme activity could be easily identified, and the shortcomings of traditional SSF could be addressed.

**Results:**

Different carbon and nitrogen sources exerted different effects on microbial growth and enzyme production. Single-factor experiments showed that glucose and yeast extract greatly improved microbial biomass accumulation and that tannin and (NH_4_)_2_SO_4_ efficiently promoted enzyme productivity. Then, these four factors were optimized through response surface methodology. Tannase activity reached 19.22 U/gds when the added amounts of tannin, glucose, (NH_4_)_2_SO_4_, and yeast extract were 7.49, 8.11, 9.26, and 2.25%, respectively. Tannase activity under the optimized process conditions was 6.36 times higher than that under the initial process conditions. The optimized parameters were directly applied to the traditional tea stalk SSF system. Tannase activity reached 245 U/gds, which is 2.9 times higher than our previously reported value.

**Conclusions:**

In this study, a modified SSF system was established to address the shortcomings of the traditional SSF system. Analysis revealed that enzymatic activity and microbial biomass are closely related, and different carbon and nitrogen sources have different effects on microbial growth and enzyme production. The maximal tannase activity was obtained under the optimal combination of nutrient sources that enhances cell growth and tannase accumulation. Moreover, tannase production through the traditional tea stalk SSF was markedly improved when the optimized parameters were applied. This work provides an innovative approach to bioproduction research through SSF.

**Electronic supplementary material:**

The online version of this article (10.1186/s13068-018-1093-0) contains supplementary material, which is available to authorized users.

## Background

Tannin acyl hydrolase (EC.3.1.1.20), commonly referred to as tannase, is an attractive biocatalyst for tannin biodegradation [1, 2]. It is extensively utilized in food [3], beverage [4], feed [5], and food additives [6], as well as in environment pollution treatments [7]. The use of tannase is particularly prevalent in the production of instant tea, acorn liquors, beers, and fruit juices [8, 9]. Although tannase is present in numerous tannin-rich plant materials, such as *Terminalia chebula* fruit, *Caesalpinia coriaria* pods, and *Anogeissus latifolia* leaves [10], it is primarily produced on a large industrial scale through microbial production to meet market demand [1]. Tannase production methods include solid-state fermentation (SSF) [11–13] and submerged liquid fermentation (SLF) [11, 14, 15]. SSF is the preferred method for tannase production because of its lower cost, lower water consumption, easier operation, and higher enzyme activity than SLF [16–18]. In SSF, tannase activity is expressed in terms of extracellular protein levels, whereas in SLF, tannase activity is expressed in terms of intracellular activity [16, 19].

Agricultural byproducts are mainly utilized as substrates in tannase production through SSF [1]. The application of agricultural byproducts in tannase production has attracted considerable attention given its advantages of low production costs and environmental friendliness. For example, Bhoite and Murthy [19] utilized a central composite rotatable design to optimize tannase production from coffee pulp through SSF. Madeira et al. [12] used castor bean residues as a solid matrix for tannase production through SSF. Beniwal et al. [20] employed rose wood (*Dalbergia sissoo*) sawdust as a substrate for tannase production through SSF.

However, numerous problems, such as complex culture components, difficult process control, and complicated microbial biomass determination methods, are encountered when agricultural byproducts are used as substrates in SSF. These problems complicate the investigation of the mechanism that underlies enzyme production in SSF. Hence, the mechanisms for effectively maintaining stable thermal mass transfer and oxygen transfer during SSF, the facile and accurate detection of microbial biomass in the solid medium, and the reduction of metabolic byproduct accumulation in the culture are important topics of SSF research.

A modified SSF method is occasionally employed to address the above concerns. In this approach, inert carriers are exclusively used as a solid matrix to absorb nutrients and to cultivate microbes. Ramos et al. [21] applied polyurethane foam as a solid support and tannin as the sole carbon source and tannase inducer. They then characterized the physicochemical properties of tannase derived from *Aspergillus niger* and obtained the kinetic and thermodynamic parameters of methyl gallate hydrolysis. Trevino et al. [22] investigated the influence of polyurethane supports on tannase production and gallic acid accumulation by *A*. *niger* Aa-20 in solid-state culture. Their results showed that compared with continuous and semidiscontinuous matrices, a discontinuous polyurethane matrix is more beneficial for tannase production and gallic acid accumulation.

Although research on SSF with inert carriers as matrices has obtained some achievements, the dynamic variety of SSF performed with chemically defined medium cannot provide sufficient theoretical guidance for optimizing the governing mechanism and parameters of SSF systems that utilize agricultural byproducts as substrates. In this case, researchers in the field of SSF have focused on the mechanisms underlying SSF systems that utilize agricultural byproducts as substrates.

The present work developed a novel optimization strategy for tannase production in a modified SSF system to solve existing problems in the traditional SSF. In this strategy, enzyme activity and biomass could be measured accurately. The measurement of these parameters revealed that the highest tannase activity, biomass, and tannase productivity could not be simultaneously obtained under the same conditions given that some factors promote cell growth, whereas others promote enzyme synthesis. Thus, optimization methods were utilized to identify the appropriate combination of process parameters that will simultaneously promote cell growth and enzyme synthesis to increase tannase production. This optimization strategy was also successfully applied in the traditional SSF to improve cell growth and tannase production. This strategy has excellent potential applications in SSF with agricultural byproducts.

## Methods

### Materials

Tea stalks were collected from a local tea-processing factory and dried at 80 °C in an oven for 2 days to a constant weight. The dried stalks were then powdered using a grinder. Polyurethane sponge (PUS) was procured from ShenLong Sponge Co., Ltd. (Fujian Province, China). Propyl gallate and rhodanine were procured from Tokyo Chemical Industry Co., Ltd. (Japan). Qualitative filter paper-201 purchased from Fushun City Civil Affairs Filter Paper Factory (China). All other chemical reagents are of analytical grade and were obtained from Sinopharm Chemical Reagent Ltd., Corp. (China).

### Microorganism and inoculum preparation

*Aspergillus tubingensis* CICC 2651 was obtained from the China Center of Industrial Collection and used to produce tannase. The strain was maintained on potato dextrose agar (PDA) slants at 4 °C. Prior to inoculation, the strain was cultivated on PDA slants at 30 °C for 4 days. Spore suspensions (1 × 10^8^ spores/mL) were then prepared by scraping spores from the surfaces of the slants into sterile physiological saline.

### Culture media and tannase production

Tea stalk powder was mixed with deionized water at the ratio of 1:20 (w/v). The mixture was heated on a heated magnetic stirrer at 80 °C for 1 h. Tea stalk extract was filtered through filter paper while still hot. PUS was used as a solid carrier and cut into a certain length, washed three times with deionized water at 60 °C, and finally washed thrice with cold water. PUS was then dried to a constant weight at 80 °C. PUS samples (1 g in weight) were placed in 250 mL Erlenmeyer flasks and autoclaved at 121 °C for 20 min. After sterilization, the PUS samples were retained in the flasks and dried to a constant weight at 80 °C. The initial culture medium (per gram PUS) for SSF comprised 6% (w/w) glucose, 6% (w/w) NH_4_Cl, and 5.6 mL tea stalk extract at the initial pH of 6.0. pH was adjusted by dilute sulphuric acid (1 mol/L) and sodium hydroxide (1 mol/L) in the initial culture solution. The medium was autoclaved at 121 °C for 20 min and subsequently cooled to room temperature. Then, 5.6 mL aliquots of the sterilized media were transferred to 250 mL Erlenmeyer flasks and thoroughly mixed with spore suspensions at the concentration of 1 × 10^8^ spores/g of PUS. The contents were incubated under 30 °C for 96 h. Changes in tannase activity and fungal biomass were detected under the initial conditions to investigate the relationship between tannase activity and fungal biomass. The morphology of microorganisms in the high-density PUS was characterized through scanning electron microscopy (SEM).

### Enzyme extraction

Crude enzyme extracts containing extracellular tannase were extracted from the fermented substrate. First, 50 mL of citrate buffer (0.05 mol/L, pH 5.0) was added to each flask. The flasks were then maintained on a rotary shaker at 25 °C and 180 rpm for 1 h. The supernatant was filtered with qualitative filter paper and the paper was washed thrice with ionized water. The supernatant was transferred to vials and stored at 4 °C for further analysis. Solids were collected from the filter paper for biomass determination.

### Enzyme assay

Tannase activity was assayed using the colorimetric method, as previously described [23]. Propyl gallate was used as the substrate for gallic acid production. Gallic acid was combined with alcoholic rhodanine to form a chromogen. The absorbance of the chromogen was detected at 520 nm using a spectrophotometer and all assays were conducted in triplicate. One unit of enzyme activity was defined as the amount of enzyme that can release 1 μmol of gallic acid per min under standard conditions. Tannase production (tannase activity on PUS) and tannase productivity (tannase activity on biomass) were expressed as units/gram dry PUS (U/gds) and units/gram dry cell weight (U/gdc), respectively.

### Biomass determination and enzyme productivity calculation

The dry PUS added to the flask was designated as *m*_1_. Filter paper predried at 80 °C to a constant weight was designated as *m*_2_. The tannase extract was filtered using dried filter paper. The paper containing mycelia and PUS was dried at 80 °C to a constant weight and designated as *m*_3_. Fungal biomass (*M*) was calculated as (*m*_3_ − *m*_2_ − *m*_1_)/*m*_1_ (mg/gds). Cell productivity (*μ*) was defined as the tannase activity (*Y*) of 1 g of dry cells and calculated as *Y*/*M* (U/gdc).

### Single-factor experiment for the adjustment of process parameters

The initial fermentation medium was used as the basal medium. Different process parameters and nutritional and growth conditions were adjusted, including the side length of PUS cubes (0.2–1.0 cm), content of tea stalk extract (75–95%), temperature of incubation (22–38 °C), initial pH of culture medium (3–7), size of inoculum (0.1 × 10^7^–25.6 × 10^7^ spores/g PUS), and type and concentration of inorganic salt (NaCl, MgSO_4_, and K_2_HPO_4_ at 0–1.5%). Among them, to keep the final concentration of nutrients consistent, different concentrations of spore suspension were prepared to inoculate in a fixed size of 1 mL. All experiments were performed in triplicate, and the mean values were reported with standard deviation.

### Additional carbon and nitrogen sources

Additional carbon and nitrogen sources have important roles in the productivity of the SSF system. To explore the influence of different carbon and nitrogen sources on tannase synthesis, the fermentation medium was supplemented with different carbon (lactose, starch, glycerol, maltose, sucrose, glucose, or tannin at 6%) and nitrogen (urea, beef extract, peptone, yeast extract, corn steep liquor, [(NH_4_)_2_NO_3_, NH_4_Cl, or (NH_4_)_2_SO_4_ at 6%] sources. All experiments were performed in triplicate and the mean values were reported with the standard deviation.

### Response surface methodology for optimizing the combination of carbon and nitrogen sources

Given their influences on biomass, tannase activity, and tannase productivity in the single-factor experiment, tannin, glucose, (NH_4_)_2_SO_4_, and yeast extract were selected for analysis and optimization through response surface methodology (RSM) [24]. The effective three-level (− 1, 0, and + 1) design (Table 1) of these parameters was selected, and 29 experiments were conducted with these levels (Table 2). The minimum and maximum ranges of the variables and the full experimental plan for RSM with respect to their values is listed in Table 2. The quadratic model for RSM for the prediction of optimal points is expressed in accordance with Eq. (1). Response surface regression analysis was conducted with the statistical software Design Expert 7.0:

1 $$\begin{aligned} Y & = \beta_{0} + \beta_{ 1} A + \beta_{ 2} B + \beta_{ 3} C + \beta_{ 4} D \\ & \quad + \beta_{ 1 1} A^{ 2} + \beta_{ 2 2} B^{ 2} + \beta_{ 3 3} C^{ 2} + \beta_{ 4 4} D^{ 2} \hfill \\ & \quad + \beta_{ 1 2} AB + \beta_{ 1 3} AC + \beta_{ 1 4} AD + \beta_{ 2 3} BC \hfill \\ & \quad + \beta_{ 2 4} BD + \beta_{ 3 4} , \hfill \\ \end{aligned}$$

**Table 1:** Variables and levels of the Box–Behnken experiment

Variables	Levels of variables			
	Code	− 1	0	1
Tannin (%)	A	3	6	9
Glucose (%)	B	3	6	9
(NH_4_)_2_SO_4_ (%)	C	6	9	12
Yeast extract (%)	D	1	3.5	6

**Table 2:** Experimental design and corresponding responses of the Box–Behnken experiment

Standard order	*A*	*B*	*C*	*D*	Tannase activity (U/gds)	
					Experimental value	Predicted value
1	3	3	9	3.5	3.63	3.73
2	9	3	9	3.5	4.13	4.84
3	3	9	9	3.5	10.07	9.01
4	9	9	9	3.5	14.60	14.15
5	6	6	6	1	12.46	11.91
6	6	6	12	1	11.88	11.57
7	6	6	6	6	8.57	8.53
8	6	6	12	6	8.60	8.81
9	3	6	9	1	8.70	8.70
10	9	6	9	1	12.80	12.80
11	3	6	9	6	6.77	6.61
12	9	6	9	6	8.92	8.76
13	6	3	6	3.5	6.49	5.48
14	6	9	6	3.5	11.93	12.38
15	6	3	12	3.5	5.68	5.06
16	6	9	12	3.5	11.90	12.75
17	3	6	6	3.5	7.24	8.12
18	9	6	6	3.5	10.07	10.34
19	3	6	12	3.5	6.95	7.19
20	9	6	12	3.5	11.60	11.23
21	6	3	9	1	6.48	7.07
22	6	9	9	1	13.57	13.84
23	6	9	9	6	3.24	3.47
24	6	9	9	6	11.38	11.30
25	6	6	9	3.5	12.89	13.38
26	6	6	9	3.5	13.79	13.38
27	6	6	9	3.5	13.75	13.38
28	6	6	9	3.5	13.09	13.38
29	6	6	9	3.5	13.35	13.38

where *Y* is the predicted response; *β*_0_ is the intercept; *β*_1_, *β*_2_, *β*_3_, and *β*_4_ are linear coefficients; and *β*_11_, *β*_22_, *β*_33_, *β*_44_, *β*_12_, *β*_13_, *β*_14_, *β*_23_, *β*_24_, and *β*_34_ are the interaction coefficients.

The responses of the dependent variables and regression analysis of experimental data were analyzed using Eq. (1). The quality of the fit of the quadratic model equation was expressed by the coefficient of determination (*R*^2^), and its statistical significance was evaluated by Fischer’s test value (*F* value).

### Traditional tea stalk SSF system

The optimized conditions of the modified SSF system were directly applied to the traditional tea stalk SSF system. The initial culture medium (per gram tea stalk) for the traditional tea stalk SSF comprised the optimized concentration of carbon sources (tannin and glucose) and nitrogen sources [(NH_4_)_2_SO_4_ and yeast extract], and 2 mL of water at pH 5.0. After sterilization, 1 mL of spore suspension was mixed with the culture medium, and the mixture was incubated under 30 °C for 96 h.

## Results

### SEM analysis of the internal structures of PUS and morphological characteristics of microorganisms

The PUS used in this experiment had a density of 40 kg/m^3^, open-hole diameter of 300–500 μm, and specific surface area of 380.6 m^2^/g. The internal structures of PUS and the morphological characteristics of *A*. *tubingensis* were observed through SEM (Additional file 1: Figure S1). The smooth, flat, and open internal structure of PUS provided extensive support and adsorption interfaces. The excellent mechanical properties of PUS ensure stable oxygen and heat transfer during SSF. Moreover, biomass and enzyme productivity could be measured conveniently with PUS as the inert carrier. The convenient measurement of these parameters is crucial for the further optimization of SSF.

### Effect of process parameters on tannase production

**Fig. 1:**
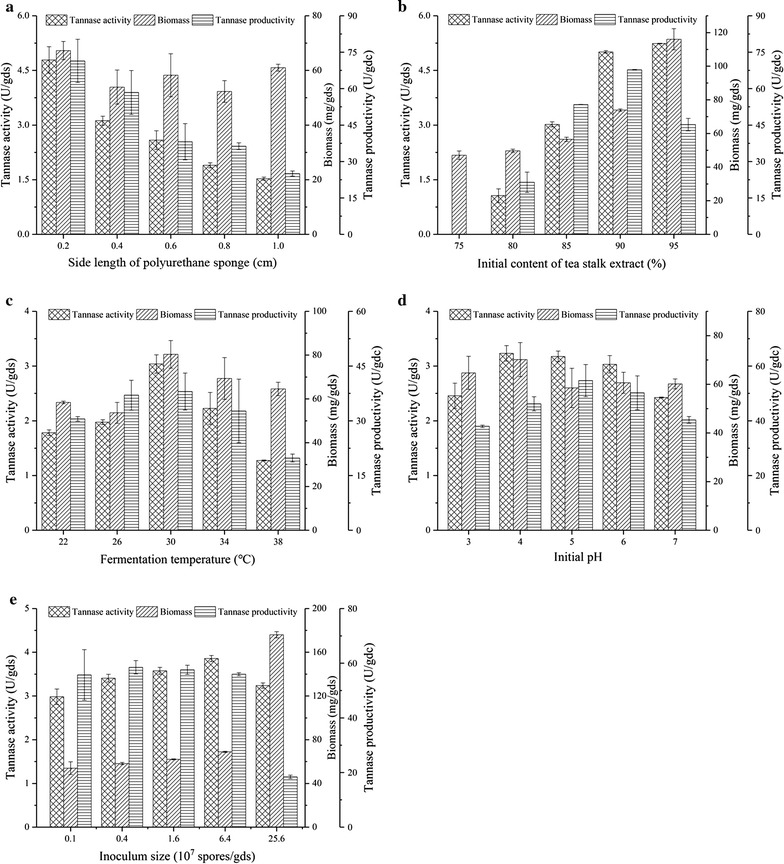
Effects of the parameters on tannase production. **a** Side length of PUS, **b** Content of tea stalk extract, **c** Temperature, **d** pH, and **e** Inoculum; the provided values are the mean ± standard deviation of three experiments

As shown in Fig. 1, different process parameters, including the side length of PUS, content of tea stalk extract, temperature of incubation, initial pH of the culture medium, and size of inoculum, were assessed, and the optimal process conditions are reshown in Table 3.

**Table 3:** Effect of different parameters on microbial growth and enzyme synthesis

Research parameters	Maximum tannase activity (U/gds)	Maximum biomass (mg/gds)	Maximum tannase productivity (U/gdc)
Side length of PUS	4.8 (0.2 cm)	67.3 (0.2 cm)	71.4 (0.2 cm)
Extract content	5.2 (95%)	116.1 (95%)	45.2 (90%)
Initial pH	3.2 (4)	70.2 (4)	54.7 (4)
Inoculum size	3.9 (6.4 × 10^7^ spores/gds)	176.0 (25.6 × 10^7^ spores/gds)	58.5 (0.4 × 10^7^ spores/gds)
Temperature	3.0 (30 °C)	80.1 (30 °C)	38.6 (30 °C)

Data in brackets denote the parameter conditions to achieve maximum microbial growth or enzyme synthesis

Some phenomena could be inferred from Fig. 1 and Table 3. First, all the investigated process parameters influenced tannase activity, biomass, and tannase productivity. Fermentation could thus be elucidated on the basis of the effects of these process parameters. Second, the highest tannase activity, biomass accumulation, and tannase productivity did not simultaneously occur under the same conditions. For example, the optimal inoculum size for tannase production is 6.4 × 10^7^ spores/g of PUS. However, biomass accumulation under this inoculum size was considerably lower than that under 25.6 × 10^7^ spores/g of PUS (Fig. 1e). Some factors promoted cell growth, whereas others promoted enzyme synthesis. This observation is common in microbial fermentation. For example, Banos et al. [25] reported that biomass does not always correspond with secondary metabolite concentration. The components of the fermentation medium can be selectively combined to promote cell growth or enzyme accumulation to achieve high enzyme production.

### Effects of additional inorganic salts

**Fig. 2:**
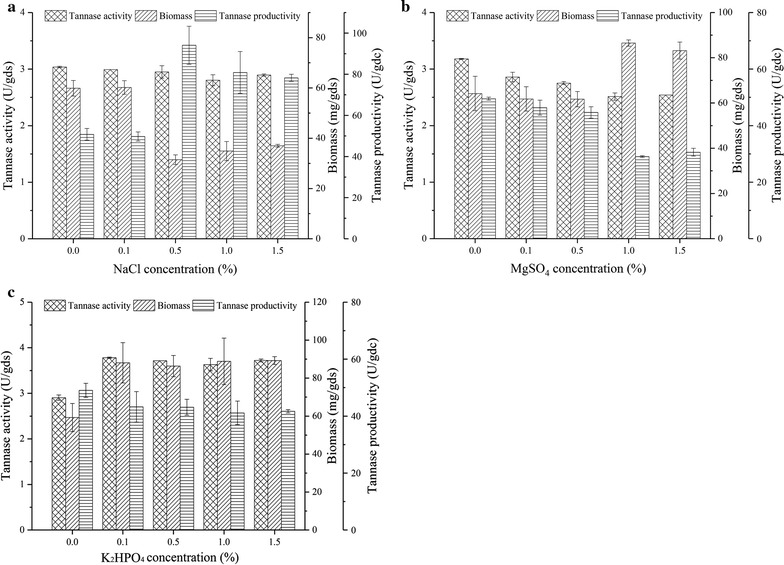
Effects of inorganic salts on tannase production. **a** NaCl, **b** MgSO_4_, and **c** K_2_HPO_4_; the provided values are the mean ± standard deviation of three experiments

The effects of different inorganic salts on tannase production were investigated. As shown in Fig. 2, NaCl did not stimulate tannase activity and instead inhibited biomass accumulation. The addition of MgSO_4_ increased microbial growth but decreased tannase activity. The addition of 0.1% K_2_HPO_4_ increased tannase production and biomass accumulation. Given that tea stalk extract likely supplied the required amount of inorganic salts for microbial growth and secondary product synthesis, the addition of inorganic salts did not significantly affect tannase production.

### Effect of individual and combined carbon sources on tannase production

**Fig. 3:**
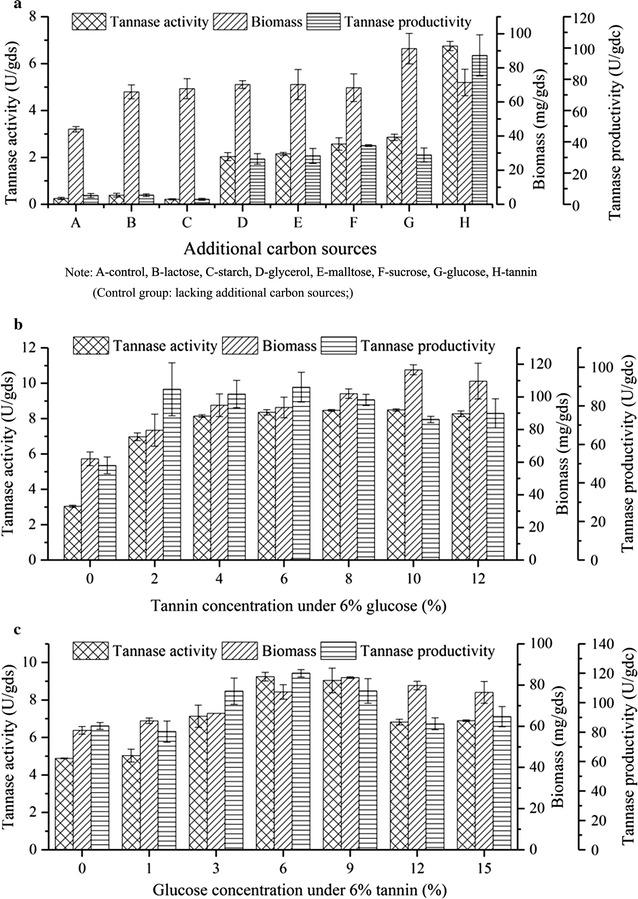
Effect of additional and combined carbon sources on tannase production. **a** Different kinds of additional carbon sources, **b** Additional tannin with 6% glucose, and **c** Additional glucose with 6% tannin; the provided values are the mean ± standard deviation of three experiments

The effect of additional carbon sources on tannase production in SSF with PUS was examined. Different kinds of carbon sources exerted different effects on cell growth, enzyme activity, and productivity. Among these carbon sources, glucose remarkably increased cell growth by 107%. The addition of tannin increased tannase activity and productivity to 6.5 U/gds and 90 U/gdc, respectively (Fig. 3a).

The effect of different tannin concentrations on tannase production with 6% glucose is shown in Fig. 3b. The initial culture medium with 6% glucose was used as the blank control. Then, the effects of tannin on tannase production were studied by adding different concentrations of tannin to the culture medium. Compared with the addition of glucose, the addition of tannin not only improved cell growth but also greatly increased tannase activity. As shown in Fig. 3b, the addition of 6% tannin increased tannase activity by 2.9 times relative to the addition of the control treatment.

Different concentrations of glucose were added to the culture medium, which contained 6% tannin. The effects of glucose concentration on cell growth and tannase production are shown in Fig. 3c. The addition of glucose improved cell growth and the highest biomass accumulation was observed in the culture medium containing 9% glucose. Cell growth and tannase activity markedly increased in the culture medium containing 6% glucose and tannin relative to those in the culture medium with tannin only, as shown in Fig. 3a. Thus, the appropriate combinations of glucose and tannin levels could increase biomass accumulation and consequently increase tannase activity and productivity.

### Effect of individual and combined nitrogen sources on tannase production

**Fig. 4:**
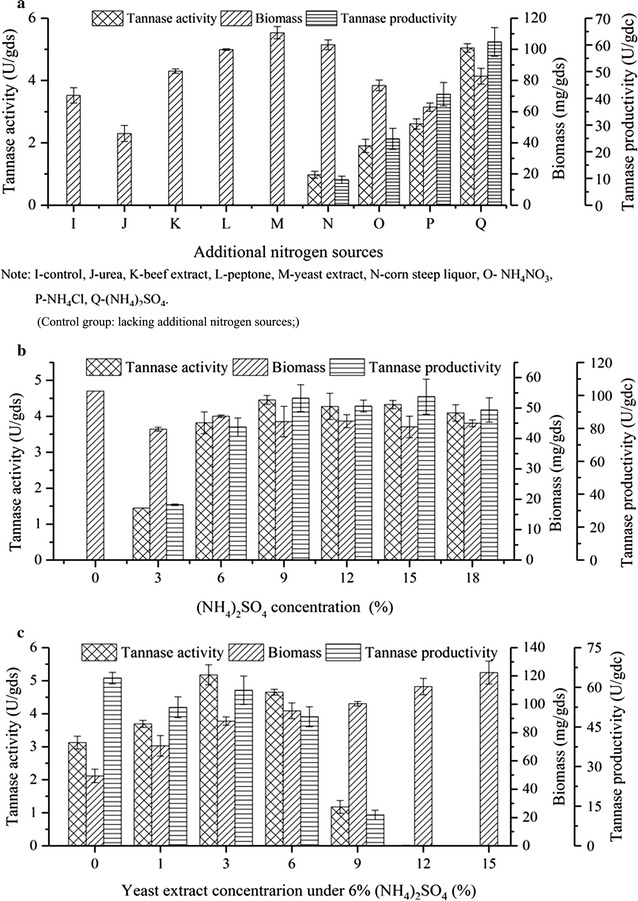
Effect of additional and combined nitrogen sources on tannase production. **a** Different kinds of additional nitrogen sources, **b** Additional (NH_4_)_2_SO_4_, and **c** Additional yeast extract with 6% (NH_4_)_2_SO_4_; the provided values are the mean ± standard deviation of three experiments

The effects of supplementation with different nitrogen sources on tannase production are shown in Fig. 4a. Different kinds of nitrogen sources had different effects on cell growth, enzyme activity, and enzyme productivity. Among the tested nitrogen sources, (NH_4_)_2_SO_4_ was the most suitable nitrogen source for tannase production. All organic nitrogen sources, except for urea, promoted biomass accumulation; in particular, yeast extract showed the most remarkable effect. Yeast extract, however, was the most unsuitable nitrogen source for promoting tannase activity. It is rarely selected as a nitrogen source for enzyme production in the traditional SSF. Nevertheless, in the present study, given that biomass could be measured accurately, we found that yeast extract could enhance cell growth.

To determine the optimal concentration of (NH_4_)_2_SO_4_, NH_4_Cl in the basal culture medium was replaced of (NH_4_)_2_SO_4_. Figure 4b shows that tannase could not be synthesized without an additional nitrogen source. (NH_4_)_2_SO_4_ drastically affected tannase activity, and the maximum tannase activity (4.5 U/gds) was observed with the addition of 9% (NH_4_)_2_SO_4_.

Different concentrations of yeast extract were added to the culture medium with 6% (NH_4_)_2_SO_4_ to investigate the effect of yeast extract on biomass and tannase production. As shown in Fig. 4c, yeast extract could significantly promote biomass production. Increasing the added amount of yeast extract also increased tannase activity. The optimal amount of added yeast extract was 3%, which increased tannase activity by 68% to 5.3 U/gds. Furthermore, compared with the addition of (NH_4_)_2_SO_4_, as shown in Fig. 4c, the addition of 3% yeast extract accelerated cell growth and did not negatively affect tannase activity. Thus, adding the appropriate concentrations of yeast extract and (NH_4_)_2_SO_4_ could increase biomass production and consequently increase tannase activity and productivity.

### Combination and optimization of carbon and nitrogen sources

The above results indicated that different carbon and nitrogen sources had different effects on enzyme synthesis and cell growth. Tannin and (NH_4_)_2_SO_4_ promoted tannase synthesis, whereas glucose and yeast extract promoted biomass accumulation. Therefore, the appropriate combination of tannin, glucose, (NH_4_)_2_SO_4_, and yeast extract at the optimal concentrations could further improve tannase activity. RSM was utilized to determine the optimal combination of these factors.

The input variables with the maximum influence on the final response (tannase activity) of the system were identified through the former experiments (Figs. 3, 4). The interactive effects of various selected factors on tannase activity [tannin, glucose, (NH_4_)_2_SO_4_, and yeast extract] were examined through RSM following central composite design (CCD). The results obtained after CCD were analyzed through standard ANOVA (Table 4), which yielded regression Eq. (2) (in terms of coded factors) for tannase activity:

2 $$\begin{aligned} Y & = 13. 38+ 1.56A + 3. 6 5B - 0.0 1 2C - 1. 5 3D \hfill \\ & \quad + 1.0 1AB + 0. 4 6AC - 0. 4 9AD + 0. 2BC + 0. 2 6BD \hfill \\ & \quad + 0. 1 6CD - 2. 5 7A^{ 2} - 2. 8 7B^{ 2} - 1. 5 8C^{ 2} - 1. 5 9D^{ 2} , \hfill \\ \end{aligned}$$

**Table 4:** ANOVA for the response surface quadratic model

Term	Tannase activity
*F* value	310.99
*P *>* F*	< 0.0001
Mean	9.81
*R* ^2^	0.9785
Adj. *R*^2^	0.9569
Pred. *R*^2^	0.8843
C.V. (%)	7.13
Adeq precision	21.227
PRESS	36.78

where tannase activity (*Y*) is a function of tannin (*A*), glucose (*B*), (NH_4_)_2_SO_4_ (*C*), and yeast extract (*D*).

To validate the obtained regression coefficient, tannase production was subjected to ANOVA, as shown in Table 5. The results of models *F* and *P *>* F* were 45.43 and < 0.0001, respectively, implying that the models are significant. The lack-of-fit values of *F* and *P* > *F* at 3.93 and 0.0994, respectively, implied that the lack-of-fit made the model fit. The coefficient of determination (*R*^2^) was 0.9785; this value indicated that the sample variation of 97.85% could be attributed to the variables and that only less than 3% of the total variance could not be explained by the model. A regression model with a *R*^2^ value higher than 0.95 has a high correlation.

**Table 5:** ANOVA results of the Box–Behnken design

Source	Sum of squares	Degree of freedom	Mean square	*F* value	*P* value *P* > *F*
Model	310.99	14	22.21	45.43	< 0.0001
*A*	29.31	1	29.31	59.95	< 0.0001
*B*	159.67	1	159.67	326.56	< 0.0001
*C*	1.752E−003	1	1.752E−003	3.583E−003	0.9531
*D*	28.23	1	28.23	57.73	< 0.0001
*AB*	4.05	1	4.05	8.28	0.0122
*AC*	0.83	1	0.83	1.70	0.2129
*AD*	0.96	1	0.96	1.96	0.1828
*BC*	0.15	1	0.15	0.31	0.5858
*BD*	0.28	1	0.28	0.56	0.4652
*CD*	0.096	1	0.096	0.20	0.6643
*A* ^2^	42.89	1	42.89	87.72	< 0.0001
*B* ^2^	53.45	1	53.45	109.31	< 0.0001
*C* ^2^	16.29	1	16.29	33.32	< 0.0001
*D* ^2^	16.31	1	16.31	33.36	< 0.0001
Residual	6.85	14	0.49		
Lack of fit	6.21	10	0.62	3.93	0.0994
Pure error	0.63	4	0.16		
Cor total	317.84	28			

Std. dev.	0.70	*R* ^2^	0.9785		
Mean	9.81	Adj *R*^2^	0.9569		
C.V. (%)	7.13	Pred *R*^2^	0.8843		
PRESS	36.78	Adeq precision	21.227		

**Fig. 5:**
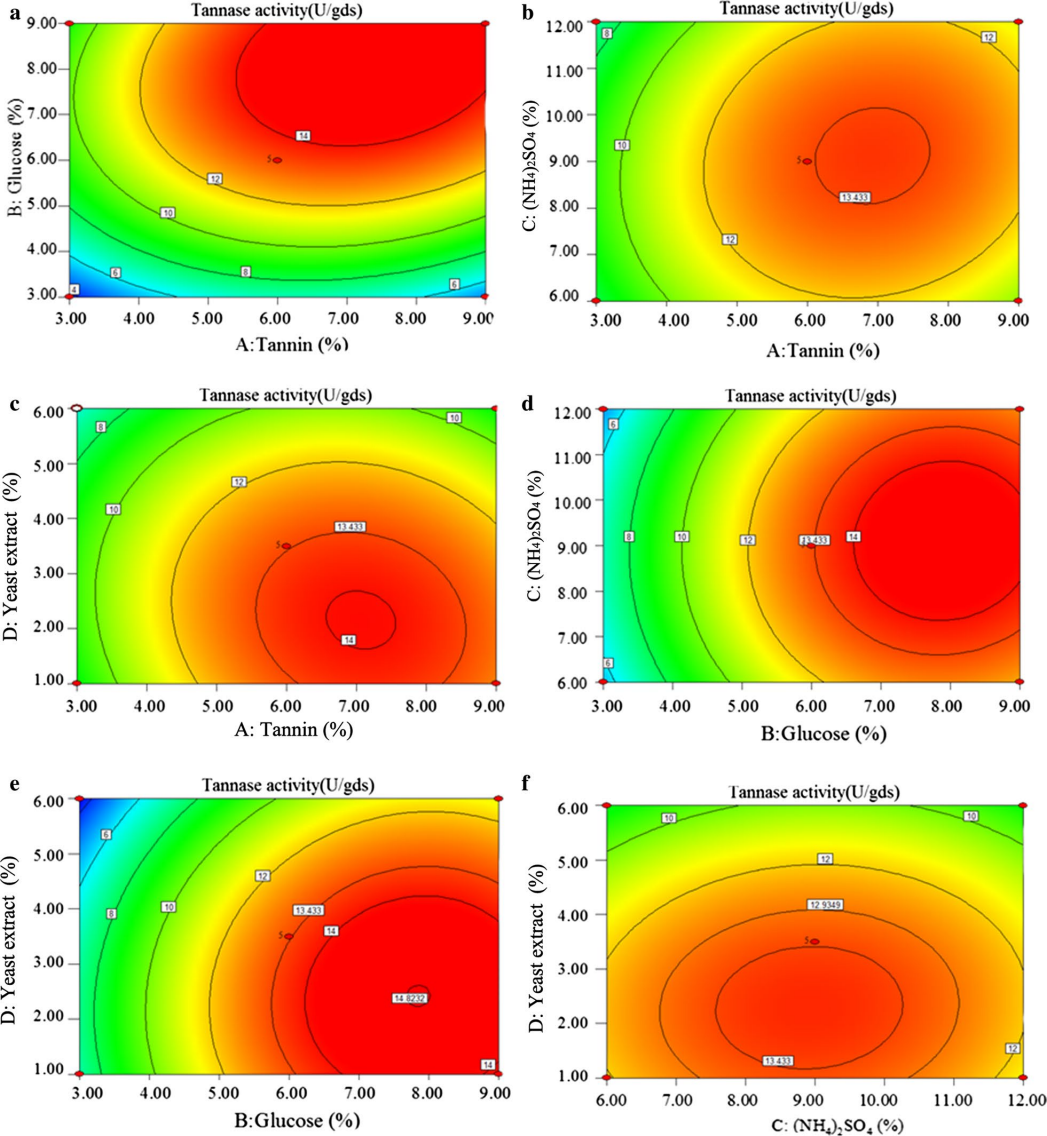
Optimization of carbon and nitrogen sources content by RSM using Box–Behnken design. **a** Interaction of tannin and glucose. **b** Interaction of tannin and (NH_4_)_2_SO_4_. **c** Interaction of tannin and yeast extract. **d** Interaction of glucose and (NH_4_)_2_SO_4_. **e** Interaction of glucose and yeast extract. **f** Interaction of (NH_4_)_2_SO_4_ and yeast extract

The influences of each variable on each response value are shown in Fig. 5. All factors interacted by different degrees, thus proving that tannase activity could be promoted by the addition of carbon and nitrogen sources at the appropriate concentrations. Moreover, the optimum tannase activity (15.43 U/gds) was observed in the SSF system with 7.49% tannin, 8.11% glucose, 9.26% (NH_4_)_2_SO_4_, and 2.25% yeast extract.

**Fig. 6:**
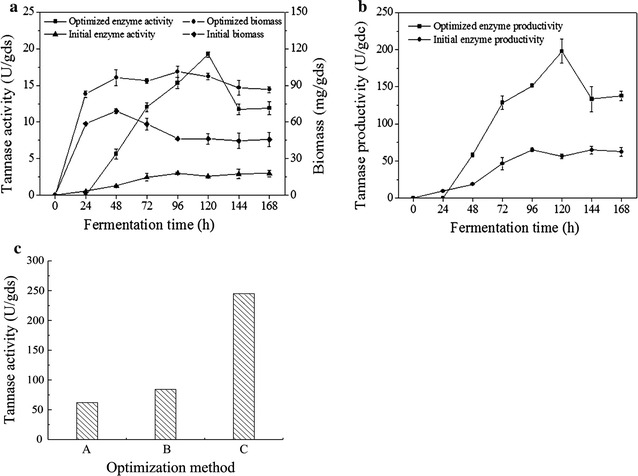
Application in modified SSF system and the traditional tea stalk SSF system. **a** Cell growth and enzyme activity in the modified SSF system. **b** Tannase productivity in the modified SSF system. **c** Comparison of tannase production reported by a previous study and that reported by the present study in the traditional tea stalk SSF system. Note: A: Maximum tannase activity under the optimized conditions with* A. niger* JMU-TS528 in a previous study [13]; B: Maximum tannase activity under the optimized conditions with* A. tubingensis* in a previous study [26]; C: Maximum tannase activity under the optimized conditions with* A. tubingensis* in the previous study

A verification test was conducted to determine the optimal conditions for SSF for tannase production. Over 168 h of fermentation, the tannase activity, biomass accumulation, and tannase productivity of the optimization group were higher than those of the initial group. These results confirmed the effectiveness of the optimized strategy (Fig. 6a, b) and verified the previous inference that energy sources with different functions could be combined to promote biomass accumulation, enzyme productivity, and enzyme activity. After 96 h of fermentation, the measured tannase activity was 15.01 U/gds, which was in accordance with the expected value of 15.43 U/gds. The maximum enzyme activity was observed at 120 h of fermentation and was approximately 19.22 U/gds, which was 6.36 times higher than that under the initial conditions (3.02 U/gds). Therefore, the proposed optimization strategy for tannase production could effectively increase enzyme activity.

### Application of the optimized process in the tea stalk SSF system

Enzyme activity is the sole index for the traditional optimization methods for SSF given that determining biomass in these methods is difficult. This index, however, is not ideal for optimization because of its limited evaluation parameters. The present results are reliable, because tannase activity and biomass could be measured accurately. The optimized process conditions for tea stalk SSF could be obtained through RSM. Moreover, the nutrient solution used in this system was extracted from tea stalk. Theoretically, the optimized process parameters could be applied in actual production.

Traditional tea stalk SSF was performed with 1 g tea stalk powder, 7.49% tannin (w/w), 8.11% glucose (w/w), 9.26% (NH_4_)_2_SO_4_ (w/w), 7.49% yeast extract (w/w), initial pH of 5.0, inoculum size of 6.4 × 10^7^ spores/gds, and fermentation time of 96 h. Tannase activity reached 245 U/gds (Fig. 6c column C), which is considerably higher than a previously obtained result (84.24 U/gds, Fig. 6c column B) [26]. Tannase activity was 2.9 times higher than that of the previously reported value. Thus, the optimization strategy is effective and could be applied to the actual tea stalk SSF system to increase enzyme activity.

## Discussion

Low-cost agricultural byproducts are widely used as substrates in SSF for the production of high-value bioproducts. However, the high insoluble substrate content of the culture medium complicates the direct determination of microbial biomass in SSF systems. Therefore, the previous studies have used the yield or activity of the target product as the sole index to evaluate or optimize SSF systems, such as those used for l(+)-lactic acid production from sugarcane bagasse [27], ethanol production from rice straw and husk [28], and rapeseed peptide production from rapeseed meal [29]. The same problem of complicated microbial biomass determination is also encountered in tannase SSF, because high amounts of agricultural byproducts, for example, castor bean residues [12], cashew testa [30], coffee husk [31], and tea stalk [26], are used as solid media to produce tannase. The previous studies have optimized fermentation conditions on the basis of a single evaluation index, including tannase activity, and ignored the effect of cell growth on tannase synthesis. Target product accumulation, however, is closely associated with biomass accumulation. In this study, we identified the optimal combination of nutrient sources that promote cell growth and tannase accumulation. Under the optimal process conditions, the maximum tannase activity reached 245 U/gds. This value is considerably higher than the maximum tannase activity of 84.24 U/gds obtained through the traditional optimized method with tea stalk as a substrate [26]. These results indicated that optimizing fermentation parameters on the basis of a single evaluation index has several limitations. As a matter of fact, the optimization of SSF with agricultural byproduct substrates still requires improvement.

In this study, the hot-water extraction method was adopted to obtain all soluble nutrients, such as tannic acid, polyphenols, polysaccharides, and other minerals, from tea stalks. The aqueous extract was then used in the modified SSF system to carry out the process of traditional tea stalk SSF. In the modified SSF system, microbial biomass and tannase productivity were taken as indexes to analyze the influence of different factors on cell growth and tannase synthesis. Afterward, critical factors, with tannase activity as an index, were selected to optimize fermentation conditions. The optimized conditions could be applied to the traditional SSF with agricultural byproducts as substrates. Interestingly, different carbon and nitrogen sources exerted different effects on microbial growth and product synthesis.

This work first analyzed the metabolic characteristics of microorganisms under the influences of different carbon sources. The carbon sources that increased cell growth and tannase synthesis were selected for process optimization. Among the tested carbon sources, glucose remarkably promoted cell growth but negligibly promoted enzyme productivity, whereas tannin efficiently improved tannase production and biomass accumulation. Although a consensus exists that glucose increases microbial cell growth [32], the role of glucose in tannase production through SSF remains controversial. For example, Beniwal et al. [20] observed that supplementation with glucose represses tannase production; however, Rodrigues et al. [33] indicated that glucose does not adversely affect enzyme production. In the traditional SSF experiments based on single-index evaluation, additional carbon sources, such as glucose that do not enhance enzyme production, are excluded from further experiments. However, the present study found that glucose increased microbial biomass accumulation. Thus, it was selected for further optimization. Moreover, tannin could enhance tannase production in SLF [34] and SSF [31, 35, 36]. This study also found that tannin could considerably increase tannase productivity by acting as an inductor and biomass production by providing energy. Therefore, when combined, glucose and tannin would complementarily promote tannase production. These two carbon sources were then selected for combinatorial optimization for the next RSM design.

The appropriate nitrogen sources for biomass accumulation and tannase synthesis were identified. Among these nitrogen sources, organic nitrogen (beef extract, peptone, yeast extract, or corn steep liquor) increased cell growth but negligibly affected enzyme synthesis. The present work discovered that yeast extract promoted biomass accumulation but repressed tannase productivity. Battestin and Macedo [31] also pointed out that yeast extract markedly decreased tannase activity but did not present data on its effect on biomass. Yeast extract is rarely used as a nitrogen source in the traditional SSF because of its negligible effect on enzyme production. Yeast extract enhanced microbial biomass accumulation in the modified SSF system and was thus included as a factor in combinatorial optimization. Inorganic nitrogen sources [(NH_4_)_2_NO_3_, NH_4_Cl, or (NH_4_)_2_SO_4_] efficiently improve tannase productivity but negligibly contribute to cell growth. Other studies have confirmed this phenomenon. For example, Zambanini et al. [37] reported that inorganic nitrogen sources negatively affect biomass accumulation. This effect consequently decreases secondary metabolite concentration. Among all the inorganic nitrogen sources used in this work, (NH_4_)_2_SO_4_ is the most suitable nitrogen source for tannase production. The highest tannase activity and tannase productivity were obtained when (NH_4_)_2_SO_4_ was used as the sole nitrogen source. Similarly, Rodrigues et al. [36] reported that among different nitrogen sources, only (NH_4_)_2_SO_4_ could improve tannase production. Thus, the addition of yeast extract and (NH_4_)_2_SO_4_ would accelerate cell growth and tannase synthesis. Therefore, these two nitrogen sources were selected as combination factors for further RSM design.

The optimal combinations of glucose, tannin, yeast extract, and (NH_4_)_2_SO_4_ were identified through RSM. Under the optimized process parameters, the maximum tannase activity reached 19.22 U/gds, which was 6.36 times higher than that under the initial conditions. Furthermore, the optimized parameters were directly applied in traditional tea stalk SSF. Tannase activity could reach 245 U/gds, which was 2.9 and 4.0 times higher than our previously reported values of 84.24 and 62 U/gds, respectively [13, 26]. The present results indicated that every nutrient in the culture medium could be comprehensively assessed with biomass and tannase productivity as indexes. Synergistic effects that further improve tannase production may be obtained when the amounts of nutrients that augment cell growth and tannase activity are balanced.

## Conclusion

A modified system was established for the rational and accurate measurement of biomass, enzyme activity, and enzyme productivity in tannase production through SSF. Results showed that different carbon and nitrogen sources exert different effects on cell growth and enzyme accumulation. Hence, tannase production could be improved by adding different combinations of different carbon and nitrogen sources that enhance biomass accumulation or enzyme productivity. The maximum tannase activity under the optimal process conditions was approximately 19.22 U/gds, which was 6.36 times higher than that under the initial conditions. The optimized process was applied in the traditional tea stalk SSF system. In this system, tannase activity reached 245 U/gds, which was 2.9 times higher than the previously reported value of 84.24 U/gds. We hope that our work provides guidance for the optimization of SSF systems that utilize agricultural byproducts as substrates.

## Additional file

**Additional file 1: Figure S1.** High-density polyurethane sponge and morphological characteristics of *Aspergillus tubingensis* CICC 2651.

## Abbreviations

SSF: solid-state fermentation
SLF: submerged liquid fermentation
PUS: polyurethane sponge
SEM: scanning electron microscope
