# Supplementary material for: Novel optimization strategy for tannase production through a modified solid-state fermentation system
Source: Biotechnol Biofuels. 2018 Apr 2;11:92. doi: 10.1186/s13068-018-1093-0 (PMC5879601; doi:10.1186/s13068-018-1093-0)
Supplement: Supplementary file 1 — Additional file 1: Figure S1. High-density polyurethane sponge and morphological characteristics of Aspergillus tubingensis CICC 2651. [file 13068_2018_1093_MOESM1_ESM.docx]

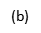

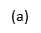

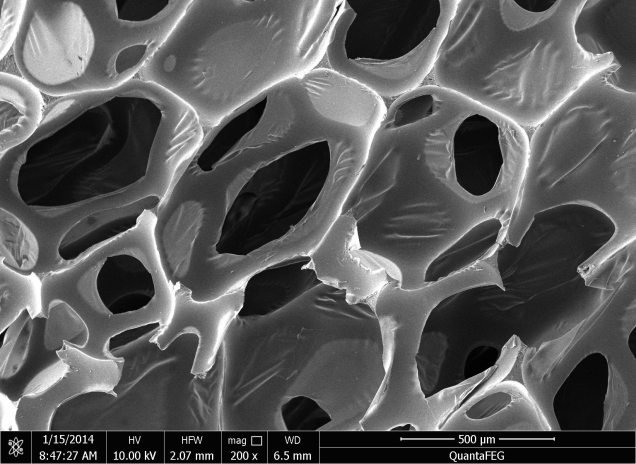

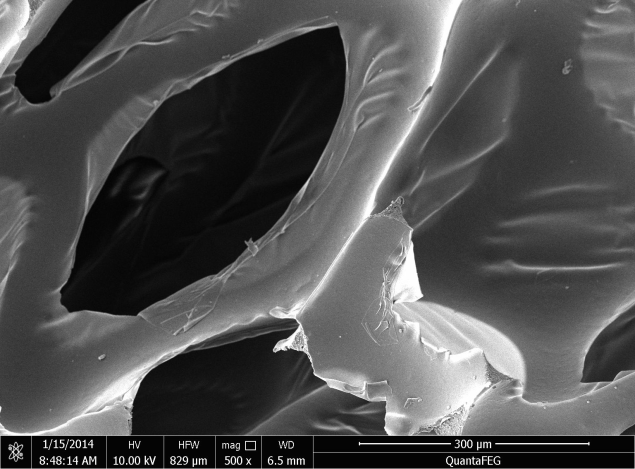


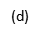

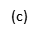

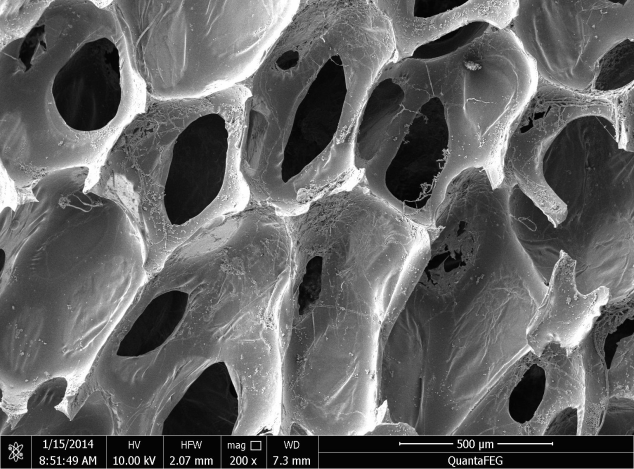

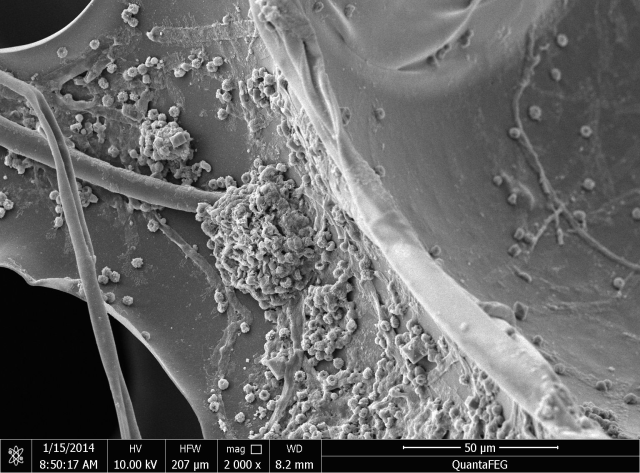


**Spores**

**Hyphae**

**Figure S1 High density polyurethane sponge and morphological characteristics of *Aspergillus tubingensis* CICC 2651. SEM images of (a) high density PUS (200×), (b) high density PUS (500×), (c) morphological characteristics of *A.tubingensis* inside of PUS (200×), (d) morphological characteristics of *A.tubingensis* inside of PUS (2000×).**
